# Localization and expression of EDS5H a homologue of the SA transporter EDS5

**DOI:** 10.1186/s12870-015-0518-1

**Published:** 2015-06-09

**Authors:** Nonglak Parinthawong, Stéphanie Cottier, Antony Buchala, Christiane Nawrath, Jean-Pierre Métraux

**Affiliations:** Department of Biology, University of Fribourg, 1700 Fribourg, Switzerland; Faculty of Agricultural Technology, King Mongkut’s Institute of Technology Ladkrabang, Chalongkrung Rd., Ladkrabang, 10520 Bangkok, Thailand; Department of Plant Molecular Biology, University of Lausanne, 1015 Lausanne, Switzerland

## Abstract

**Background:**

An important signal transduction pathway in plant defence depends on the accumulation of salicylic acid (SA). SA is produced in chloroplasts and the multidrug and toxin extrusion transporter ENHANCED DISEASE SUSCEPTIBILITY5 (EDS5; At4g39030) is necessary for the accumulation of SA after pathogen and abiotic stress. EDS5 is localized at the chloroplast and functions in transporting SA from the chloroplast to the cytoplasm. EDS5 has a homologue called EDS5H (EDS5 HOMOLOGUE; At2g21340) but its relationship to EDS5 has not been described and its function is not known.

**Results:**

EDS5H exhibits about 72 % similarity and 59 % identity to EDS5. In contrast to EDS5 that is induced after pathogen inoculation, EDS5H was constitutively expressed in all green tissues, independently of pathogen infection. Both transporters are located at the envelope of the chloroplast, the compartment of SA biosynthesis. EDS5H is not involved with the accumulation of SA after inoculation with a pathogen or exposure to UV stress. A phylogenetic analysis supports the hypothesis that EDS5H may be an H^+^/organic acid antiporter like EDS5.

**Conclusions:**

The data based on genetic and molecular studies indicate that EDS5H despite its homology to EDS5 does not contribute to pathogen-induced SA accumulation like EDS5. EDS5H most likely transports related substances such as for example phenolic acids, but unlikely SA.

**Electronic supplementary material:**

The online version of this article (doi:10.1186/s12870-015-0518-1) contains supplementary material, which is available to authorized users.

## Introduction

The signal transduction for induced resistance to many pathogens including viruses, bacteria, fungi and oomycetes involves the phenolic compound salicylic acid (SA) [1–3]. The importance of SA for the activation of defences has been repeatedly demonstrated with a number of mutants or transgenic plants impaired in the accumulation of SA. In particular, the *Arabidopsis thaliana eds5/sid1* (*enhanced disease susceptibility5/salicyclic acid-deficient 2*) and *sid2* mutants accumulate only 10 % of the SA produced in wild-type plants after induction and exhibit increased susceptibility to *Pseudomonas syringae* and *Hyaloperonospora parasitica* and fail to express the pathogenesis-related gene *PR1* [4]. The *eds5/sid1* mutation was found in a gene encoding a member of the MATE (*MULTIDRUG AND TOXIN EXTRUSION*) transporter family [1], while the *sid2* mutation was found in the *ISOCHORISMATE SYNTHASE1* gene (*ICS1*) [5].

The identification of ICS1 in *A. thaliana*, definitely demonstrated the importance of the isochorismate pathway for SA biosynthesis, similar to the pathway described in some *Pseudomonas* species [5]. The ICS1 gene product was confirmed to possess ICS activity and to be targeted to the plastidic compartment [6]. Synthesis of SA following exposure to ozone in *Arabidopsis* was also proposed to proceed through the activity of ICS enzymes [7]. The involvement of isochorismate in the synthesis of SA was confirmed in transgenic tobacco plants overexpressing an ICS of *Catharanthus roseus* [8] as well as in tomato [9] and *Nicotiana benthamina* [10]. The second *ICS* gene present in the *Arabidopsis* genome named *ICS2* [5] encodes a protein that is also localized in the chloroplast and has ICS activity [11]. ICS2 participates in the synthesis of SA in partial redundancy with ICS1 since *ics1/ics2* double mutants produce only 36 % of the SA amount found in the *ics1* single mutant. The conversion from isochorismate to SA has not yet been described in plants. In *Pseudomonas aeruginosa* it is catalyzed by a bifunctional enzyme displaying isochorismate pyruvate-lyase and chorismate mutase activities [12]. Thus, at the current state of knowledge, more than 95 % of SA synthesized under inductive conditions is formed in the chloroplast.

Interestingly, a recent analysis showed that EDS5 is localized at the chloroplast envelope and functions in the export of SA from the chloroplast to the cytoplasm [13] [14]. It represents one of the few regulated transporters involved in the movement of a signal for induced defences.

In fact, MATE-transporters are present in almost all prokaryotes and eukaryotes and are thus one of the most conserved families in nature [15, 16]. Plants have the largest gene family of MATE-transporters with 58 genes in *Arabidopsis*, while prokaryotes have approximately 10 genes and mammals only 2. Plant MATE–transporters have not only been found at the plasma membrane, but also at the vacuolar membrane acting in the sequestration of toxic metabolites. For example, the MATE-transporter TT12 acts as flavonoid/H^+^ antiporter at the vacuole and is active in proanthocyanidin accumulation in the seed coat of *Arabidopsis* [17, 18]. In tobacco, the alkaloid nicotine is sequestered into the vacuole in exchange with protons by the action of NtMATE1 and NtMATE2 in the roots and by the MATE-transporter NtJAT1 in the shoots [19, 20]. These MATE-transporters may also transport other alkaloids, such as anabasine, hyoscyamine, scopolamine or berberine, but no flavonoids. Here we report a detailed characterization of EDS5H, a close homologue of the SA transporter EDS5.

## Results

### Identification and characterization of EDS5H

**Fig. 1:**
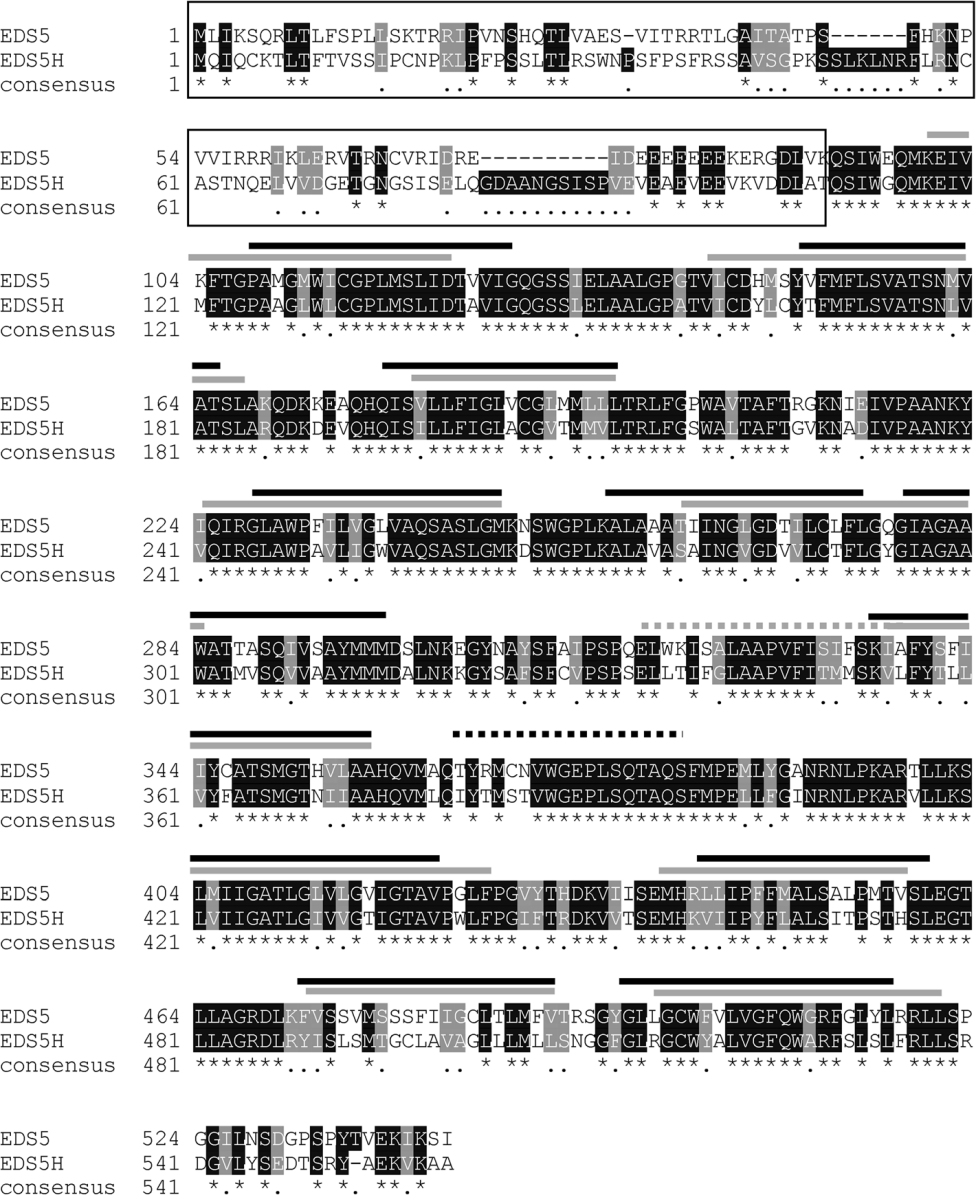
Alignment of the predicted protein sequences of EDS5H and EDS5. The predicted membrane-spanning domains are indicated above the alignment with a grey bar for EDS5H and black bars for EDS5. Identical amino acids are indicated with an asterisk, and conserved amino acids are indicated with a single dot. The N-terminus region has a low degree of homology and is indicated by a black frame

The analysis of the Arabidopsis genome revealed a homologue of EDS5 that is encoded by the gene At2g21340 and was therefore named EDS5H. The *EDS5H* gene was characterized by amplification of a 1680 bp cDNA by reverse transcriptase-mediated polymerase chain reaction (RT-PCR) and subsequently sequenced. The analysis of the *EDH5* sequence confirmed that *EDS5H* has an open reading frame (ORF) of 1680 bp encoding for a protein of 559 amino acids. The genomic region of *EDS5H* consists of 14 exons and 13 introns based on the annotated Arabidopsis genome. The alignment between the predicted protein sequences of EDS5 and EDS5H showed an overall 72 % similarity and 59 % identity. However, the 100 aa at the N-terminus showed less conservation (20 % identity) (Fig. 1).

### Expression of EDS5H

**Fig. 2:**
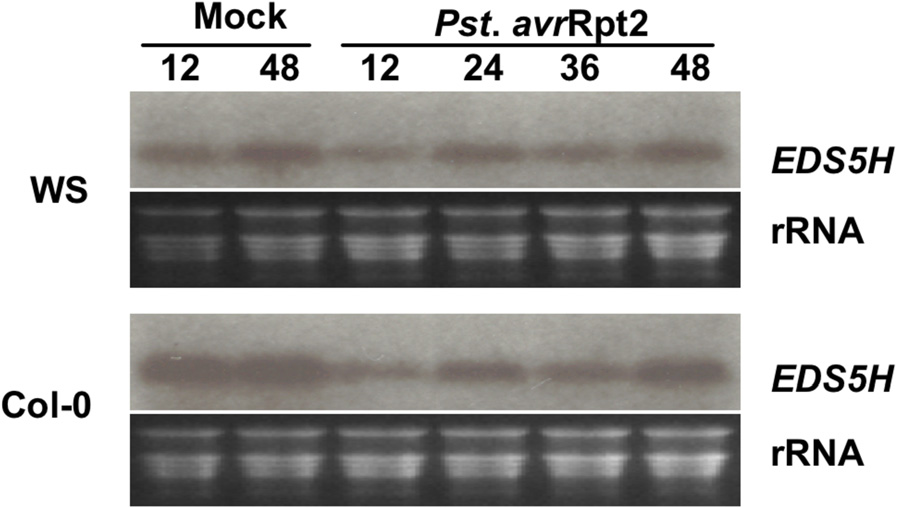
Expression analysis of EDS5H. *A. thaliana* plants (Col-0 and Ws) were inoculated with *P. syringae* pv. *tomato avrRpt2* (*Pst*) and harvested for RNA extraction at 12, 24, 36, and 48 h after inoculation. Mock control was carried out by treatment with 10 mM MgCl_2_. The RNA blot was probed with a gene-specific probe for *EDS5H*. Ethidium bromide staining of the RNA gel (rRNA) was used to show equal loading. The experiment was repeated 3 times with similar results

**Fig. 3:**
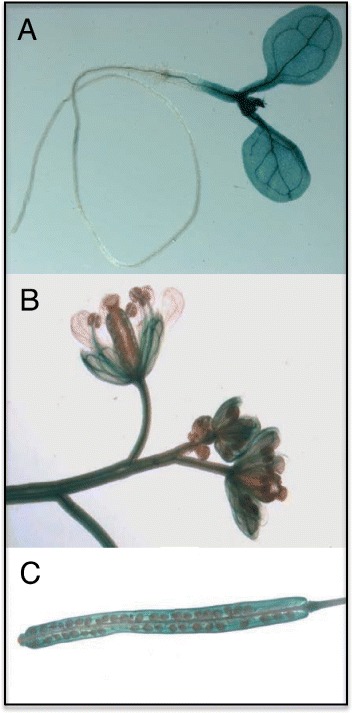
Localization of GUS activity in transgenic Arabidopsis plants expressing the pEDS5H::GUS fusion construct. Seedlings, siliques or inflorescence parts of plants transformed with EDS5H::GUS constructs were stained with the substrate for GUS. The activity of a promoter of 2000 bp length is shown in a seedling **(a)**, flowers **(b)**, and a silique **(c)**. The experiment was repeated 3 times with similar results

The expression of *EDS5H* was studied in leaves of plants inoculated with *Pseudomonas syringae* pv *tomato avrRpt2*. As indicated in Fig. 2, the expression shows no change in response to infection. The activity of the promoter of *EDS5H* was also studied using promoter-*GUS* fusions. Promoter fragments of 500, 1000 or 2000 bp length were fused to *GUS*. Transgenic Arabidopsis plants carrying *pEDS5H::GUS* fusions of different lengths all gave the same activity pattern. The *EDS5H* promoter was very active in all green tissues but not in roots or in petals (Fig. 3).

### Subcellular localization of EDS5H

**Fig. 4:**
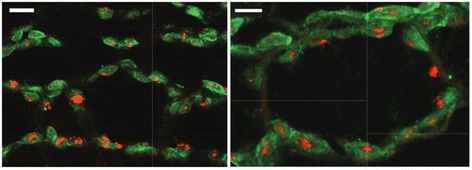
Subcellular localization of EDS5H-3myc in transgenic Arabidopsis. Mesophyll cells of transgenic plants carrying the CaMV35S::EDS5H::3xmyc construct. The two panes show the cMyc-epitope labelled with Alexa Fluor 488 excited at the wavelength of 488 nm and detected using the emission filter 522 DF32 (green) and Red chlorophyll catalase reductase labelled with Alexa Fluor 568 excited at a wavelength of 568 nm and detected using the emission filters 605 DF32 and 585 EFLP (red). Bar = 5 μm. A representative picture out of 11 transformed lines is presented

The ORF of *EDS5H* was placed under the control of the CaMV 35S promoter upstream of a triple cMyc epitope tag. In tissue sections of transgenic plants carrying the CaMV35S::EDS5H::3xcMyc construct, the cMyc-epitope tag was labelled with a primary anti-myc antibody that was identified by a goat anti-mouse IgG conjugated with Alexa Fluor 488 (green). RCCR (red chlorophyll catalase reductase) was used as an example for a protein targeted to the chloroplast [21]. Tissues were labelled with a primary antibody against RCCR and secondary antibody goat anti-rabbit IgG conjugated with Alexa Fluor 568 (red). Fig. 4 shows an intense and fine green labelling localized around the chloroplast, while the red label was in the middle of the chloroplast. The co-localization of the EDS5H with RCCR shows that EDS5H is located to the chloroplast. cMyc-tag could not be detected in plant cells when the natural *EDS5H* promoter was used, although the transcription of the EDS5H::cMyc could be determined by RT-PCR (data not shown). This suggests that the *EDS5H* promoter, which is constitutive, was too weak to drive the whole cassette of the *EDS5H*::3xcMyc to its target organelle or the amount was below detection limits. Taken together, these studies show that EDS5H is localized at the chloroplast as was shown previously for EDS5 [11].

### Analysis of plants with downregulated *EDS5H* expression

**Fig. 5:**
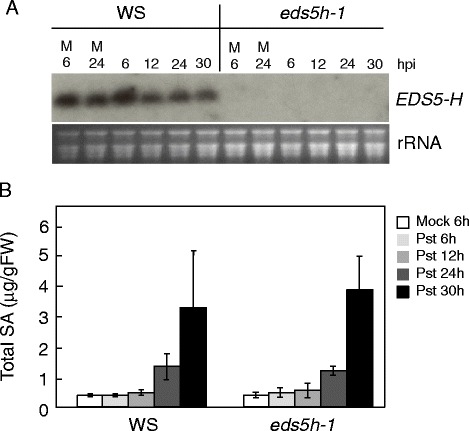
Characterization of the *eds5h-1* mutant. **a)** Expression analysis of the *EDS5H*. 10 mM MgCl_2_ (M) or *P. syringae* pv. *tomato* DC3000 were infiltrated into Ws and *eds5h*-1 leaves. Samples were taken at 6, 12, 24, and 30 h post-inoculation (hpi). Northern blots were hybridized with a gene-specific probe for *EDS5H*. Ethidium bromide staining of the RNA gel (rRNA) was used as control for the loading. The experiment was repeated 3 times with similar results. **b)** Accumulation of SA in plants infiltrated with 10 mM MgCl_2_ (M) or *P. syringae* pv*. tomato* DC3000 (Pst); leaves were harvested 6, 12, 24, and 30h hpi. For each time point n = 3 (±SD), the experiment was repeated 3 times

**Fig. 6:**
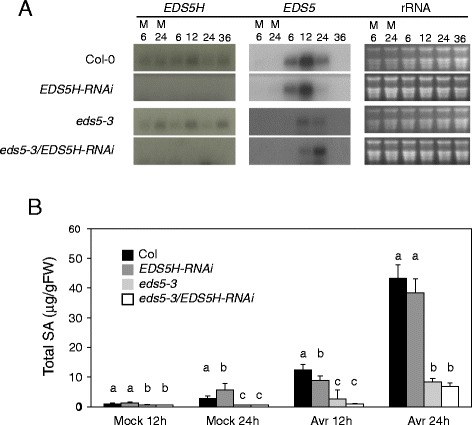
Characterization of the *EDS5H-RNAi* mutant and *eds5-3/EDS5H-RNAi* double mutant. **a)** Expression analysis of *EDS5H* and *EDS5* in different genotypes. Plants were inoculated with 10 mM MgCl_2_ (M) or *P. syringa*e pv. *tomato* DC3000. Samples were taken at 6, 12, 24, and 36 hpi. Northern blots were hybridized with gene-specific probes for *EDS5H* and *EDS5*. Ethidium bromide staining of the RNA gel (rRNA) was used as loading control. Note that some *EDS5* expression can be detected as *eds5-3* is not a transcriptional null mutant. The experiment was repeated 3 times with similar results. **b)** Accumulation of SA in different genotypes. Plants were inoculated with 10 mM MgCl_2_ (Mock) or *P. syring*ae pv. *tomato* DC3000 carrying *avrRpt2* (Avr). Samples were taken 12 and 24 hpi. Different letters above each bar represent statistically significant differences. The mean comparison of total SA was analyzed using Duncan’s multiple-range test (DMRT) with p-value ≤ 0.05. For each time point n = 4 (±SD), the experiment was repeated 3 times

Since both EDS5 and EDS5H are expressed in the same tissues and in the same organelle it is possible that EDS5H may also function in SA accumulation since it has been observed that *eds5-3* mutants still contain ca 10 % of the SA level found in wild-type plants [2]. This level of SA could potentially be due to the functional EDS5H. A mutant carrying a T-DNA insertion in the *EDS5H* gene, called *eds5h-1*, was isolated after screening the population of T-DNA insertion pools generated by the Arabidopsis knock-out facility service at the University of Wisconsin-Madison. Two mutant lines were obtained, *eds5h-1* carrying the T-DNA insertion at 229 bp (exon 1) and *eds5h-10* carrying the insertion at 1360 bp (exon 10). Both were transcriptional null alleles, and the *eds5h-1* mutant was used for further studies. The expression of *EDS5H* in Ws wild-type plants was strong in non-inoculated leaves (Fig. 5a). The expression of *EDS5* was not different between wild type and the *eds5h-1* mutant (data not shown). No difference was detectable in the accumulation of SA after inoculation with *P. syringae pv. tomato* DC3000 in Ws and *eds5h*-*1*, most likely due to EDS5 function (Fig. 5b). Since the induction kinetics of SA accumulation in Col-0 and Ws showed strong differences, the generation of a double mutant between *eds5h-1* (in the Ws background) and *eds5-3* (in the Col-0 background) was not useful (data not shown). Therefore, we used RNA interference (RNAi) to downregulate the transcription of *EDS5H* in the Col-0 background. For the construction of the RNAi construct, the 350 bp 5’-non-conserved region of *EDS5H* was cloned into the plasmid pHANNIBAL [22] in sense and antisense orientations. The RNAi construct was transformed into both Col-0 and *eds5-3*. Independent lines from *eds5h* (*EDS5H-RNAi*) and from the double mutant *eds5-3/eds5h* (*eds5-3*/*EDS5H-RNAi*) were selected for further investigation because they did not accumulate *EDS5H* mRNA (Fig. 6a). These lines showed no changes in phenotype compared to wild-type plants. Transgenic plants in which no *EDS5H* transcription was detected after infection with *P. syringae pv. tomato* DC3000 with or without the avirulence gene *avrRpt2* were analysed for their SA content in comparison to Col-0 plants. A slight decrease in SA accumulation (ca 20 %) was observed in *EDS5H-RNAi* plants 12 h after infection with *P. syring*ae pv. *tomato* DC3000 *avrRpt2* (Avr) compared to Col-0 but this difference disappeared 24 h after infection. Similarly, no changes were observed when EDS5H was knocked down by RNAi in the *eds5-3* mutant (Fig. 6b). Similar results have been found for the SA accumulation after UV-light exposure (data not shown). These results support that *EDS5H* is not responsible for the presence of the residual SA in the *eds5-3* mutant.

### Analysis of transgenic *eds5-3* plants overexpressing *EDS5H* and a *EDS5::EDS5H* fusion protein

**Fig. 7:**
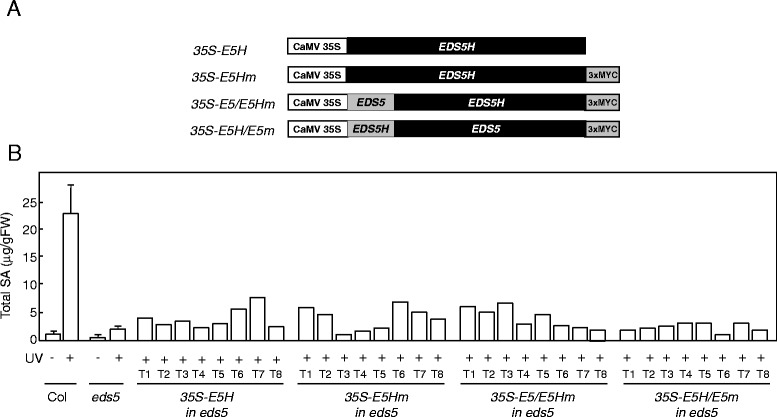
Characterization of transgenic *eds5-3* plants carrying constructs for the overexpression of *EDS5H* or *EDS5H* variants. **a)** Schematic representation of the constructs used. **b)** Accumulation of total SA in different genotypes overexpressing *EDS5H* and its variants. Plants were exposed to UV-C light for 20 min and samples were taken 12 h later. For determinations with Col0 and *eds5,* each time point n = 3 (±SD), SA determinations on all transformants were carried out once in the T1 generation

In order to determine if overexpression of *EDS5H* can rescue the SA-deficient phenotype of *eds5-3*, *EDS5H* was expressed in *eds5-3* under the control of the 35S promoter (Fig. 7a). The lines overexpressing EDS5H did not display any phenotypic change compared to wild types. Transgenic plants overexpressing *EDS5H* were exposed to UV-light to induce the SA-biosynthesis pathway and their SA content was measured 12 h after induction. Some of the independent *eds5-3* lines overexpressing *EDS5H* accumulated up to 3 times more SA than the *eds5-3* controls but still 5 times less than the wild-type plants (Fig. 7b). The rescue of the *eds5-3* mutant phenotype by the *CaMV35S::EDS5H* construct was therefore only limited.

EDS5 and EDS5H show a very high percentage of homology at the C-terminal region containing the transmembrane spanning domains. In contrast, a small domain of approximately 60 aa that lies between the signal peptide for plastid targeting and the beginning of the transmembrane spanning domains shows little homology. In EDS5, this domain contains a hepta-peptide potentially forming a coiled-coil domain (http://embnet.vital-it.ch/software/COILS_form.html), while not in EDS5H. In order to test if the N-terminal domain confers specificity to the function of EDS5, *eds5-3* plants were transformed with a CaMV35S::EDS5-EDS5H fusion that contained the plastid localization signal from EDS5 as well as the N-terminus of the mature protein. The accumulation of SA was monitored in transgenic *eds5-3* plants overexpressing the EDS5::EDS5H fusion protein after their exposure to UV-light followed by a 12-h incubation time. The expression of the EDS5::EDS5H fusion protein did not result in a better rescue of the *eds5-3* phenotype than the original EDS5H (Fig. 7b), but still increased the SA amount of the *eds5* mutant suggesting that the resulting fusion protein still had transport activity. The fusion of the entire N-terminal domain of EDS5H to the EDS5 transporter region did not rescue SA accumulation (Fig. 7b). These results show that EDS5H does not function better in SA accumulation when the EDS5 N-terminus is present.

### Potential transport function of EDS5 and EDS5H

**Fig. 8:**
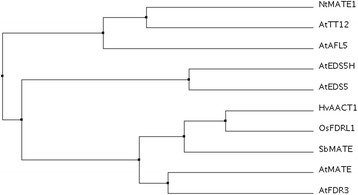
Phylogenetic studies of the MATE-family of plants. *EDS5* and *EDS5H* have been aligned in comparison to plant MATE shown to transport flavonoids (AtTT12) [18], alkaloids (NtMATE) [20] or molecules such as polyvinylpyrrolidone and pyrrolidinone (AtAFL5) [25], as well as citrate transporters from monocots (barley [26], sorghum [27] and rice [28]) and Arabidopsis (AtFRD3 [29] and AtMATE [24]). The dendrogram was established with the ClustalW program

Phylogenetic studies of the MATE-family of plants revealed that EDS5 and EDS5H are two transporters that are in the same subfamily of MATE-transporters as the *Arabidopsis* citrate transporters described recently [23, 24]. Therefore, EDS5 and EDS5H have been aligned in comparison to plant MATE transporters that have been shown to transport flavonoids (AtTT12) [18], alkaloids (NtMATE) [20] or other toxic molecules, such as polyvinylpyrrolidone and pyrrolidinone (AtAFL5) [25], as well as citrate transporters from monocots (barley [26], sorghum [27] and rice [28]) and Arabidopsis (AtFRD3 [29] and AtMATE [24]). This alignment shows that EDS5 and EDS5H form their own subgroup clustering together with the citrate transporters and not with the flavonoid/alkaloids/toxin transporters (Fig. 8). Since EDS5 was shown to transport SA, EDS5H might transport related phenolic compounds, but unlikely SA.

## Discussion

A detailed characterization of the related MATE-transporter EDS5H was undertaken in order to obtain more information on its possible function relative to EDS5. The present studies confirm earlier results that EDS5 is only weakly expressed under non-inducing conditions and strongly after pathogen infection as reported for SA accumulation (Fig. 6a) [1]. The homologue EDS5H is constitutively expressed in all green tissues, independently of pathogen infection (Fig. 3). The fact that the *eds5-3* mutant phenotype is clearly detectable under conditions where EDS5H is strongly expressed indicates that EDS5 and EDS5H may only have partially redundant functions.

The determination of the sub-cellular localization of EDS5H by fluorescence microscopy reveals that it is localized at the plastid where most of the SA is synthesized [13, 30]. This is similar to EDS5 that was shown to localize within the chloroplast envelope and to function as a multidrug and toxin extrusion-like transporter in the export of SA from the chloroplast to the cytoplasm where it controls the innate immune response [13].

In order to clarify the functional relationship between EDS5 and EDS5H, T-DNA insertion mutants and RNAi lines where the *EDS5H* transcript was knocked out have been isolated or produced in the Col-0 or in the *eds5-3* mutant background. The analysis of all these lines clearly show that EDS5H is not involved in SA accumulation after pathogen or UV-light induction (Fig. 6). Two explanations may be proposed for why the *eds5-3/eds5H* double mutants do not have less SA than *eds5-3*. Firstly, EDS5H could have a very low SA transport activity and therefore not reduce the SA content in the plastid. Secondly, the SA content that remains in the *eds5-3* mutant is actually not produced in the plastid. Similarly, the single mutant *ics1* or the double mutant *ics1/ics2* contain a similarly low amount of SA indicating the existence of an alternative pathway for the production of SA [11]. Also, *eds5-3* lines overexpressing EDS5H contain only slightly more SA independent of whether EDS5H was equipped with the N-terminus of EDS5 or not. These studies suggest that EDS5H has no, or at best a very weak transport activity for SA and is not involved in resistance to *P. syringae* pv. *tomato* or *Botrytis cinerea* (unpublished data). Since EDS5H is closely related to EDS5 (Fig. 8) it is most likely involved in transporting related substances, for example other phenolic acids. The biochemical characterization of the transport activities of EDS5H will be exciting objectives for future work.

## Conclusions

Despite its homology to EDS5, EDS5H does not contribute to pathogen-induced SA accumulation like EDS5. The phylogenetic relatedness of EDS5H with EDS5 supports a function of EDS5H in the transport of phenolic substances.

## Materials and methods

### Growth conditions, bacterial inoculations and plant transformation

Arabidopsis accessions Columbia-0 (Col-0) and Wassilewskija (Ws) or transgenic plants were grown under either sterile or non-sterile conditions. Non-sterile plants were grown on a pasteurized soil mix of commercial potting soil:perlite (3:1), in a growth chamber at 22 ± 2 °C under a 12-h photoperiod. Seed dormancy was broken by stratification at 4 °C for 3 days. For sterile cultures, seeds were surface sterilized by treating for 15 min in 2.5 % (v/v) commercial bleach containing 0.05 % Triton X-100 with continuous agitation and rinsed four times with sterile distilled water. Seeds were placed on solid medium consisting of ½ MS (Murashige and Skoog basal medium, Sigma) with 1 % sucrose, 0.1 % vitamins (Murashige and Skoog, Sigma). *P. syringae* pv *tomato* with or without *avrRpt2* was cultured at 28 °C and 220 rpm in Luria-Bertani medium containing 30 μg/ml rifampicin and 50 μg/ml kanamycin. Four-week-old plants were syringe-inoculated with a suspension of ~ 10^5^ colony-forming units per ml in 10 mM MgCl_2_ and mock control plants with 10 mM MgCl_2_.

Arabidopsis was transformed by the floral dip method [31]. *A. tumefaciens* strain GV3101, transformed by heat shock with the construct of interest, was grown at 28 °C in LB medium with an appropriate antibiotic. Transgenic plants were selected on ½ MS plates, containing 30 μg/ml hygromycin.

### Cloning of EDS5H and sequence analysis

cDNA of *EDS5H* (At2g21340) was amplified from Col-0 plants using the Access RT-PCR system from Promega. The 5’ end of the cDNA was determined by RT-PCR using primers located 75 (75UpStream), and 150 (150UpStream) bp upstream of the predicted ATG on the annotated genomic sequence (for these and all other primers indicated below, see sequences in Additional file 1: Figure S1). The 3’ end of the cDNA was determined by RT-PCR using primers located 50 (50DownStream), 100 (100DownStream), 200 (200DownStream), 250 (250DownStream), and 300 (300DownStream) bp downstream of the stop codon of the annotated genomic sequence. RT-PCR products were cloned into pGEM-T Easy Vector (Promega) for sequencing.

### Isolation of RNA and DNA, transcript analysis

For RNA gel blot analysis, RNA was isolated as previously described [4]. For qPCR, RNA was isolated using the Qiagen RNA easy kit including the recommended DNAase treatment. Genomic DNA was isolated using a protocol modified from [32]. Briefly, a leaf was extracted with buffer (0.2 M Tris–HCl pH 9.0, 0.4 M LiCl, 25 mM EDTA, 1 % SDS). The extracted tissue was centrifuged and the DNA was precipitated with isopropanol. The airdried pellet was resuspended in TE (10 mM Tris pH 8.0, 1 mM EDTA).

For RNA gel blot analysis, total RNA (10 μg) was separated in formaldehyde-agarose (1 %) gels, transferred to a Nylon membrane (Hybond-N, Amersham Biosciences, UK) and crosslinked by UV-light. Hybridization was carried out in hybridization buffer (0.5 M NaHPO_4_ pH 7.2, 7 % SDS, 1 mM EDTA, 1 % BSA) at 65 °C. The membrane was washed twice with 2X SSC containing 0.1 % SDS and twice with 0.2X SSC containing 0.1 % SDS at 65 °C, before exposing to X-Omat or Bio Max film (Kodak).

### Isolation of mutants carrying a T-DNA in the *EDS5H* region

Plants carrying a T-DNA fragment in the region of exon 1 and exon 10, called *eds5h-1* and *eds5H-10*, respectively, were screened by PCR www.biotech.wisc.edu/NewServicesAndResearch/Arabidopsis. *eds5H-1* plants were isolated by using the forward primer JL-202, which is present on the left border of T-DNA region (JL-202) and the reverse primer GSP1. The homozygous line was selected by the absence of the wild-type fragment using GSP2 and GSP1 as forward and reverse primers, respectively. *eds5h-10* plants were selected by using JL-202 as forward primer and GSP3 as reverse primer. A homozygous line was identified by the absence of the wild-type fragment by using GSP4 and GSP3 as forward and reverse primers, respectively.

### Construction of the plasmid used for RNAi

A fragment of about 350 bp of the non-conserved region of *EDS5H* was amplified by RT-PCR and cloned into plasmid pHANNIBAL [22] in sense and anti-sense orientation. The sense strand was designed to include *Xho*I and *Kpn*I as cloning sites (forward primer: E5H-sens-For; reverse primer: E5H-sens-Rev) and the anti-sense strand was designed to include *Bam*HI and *Cla*I as cloning sites (forward primer: E5H-anti-For; reverse primer: E5H-anti-Rev). The construct was cloned into the binary vector pART27 using the *Not*I sites [33].

### Construction of promoter:GUS and cMyc-tagged constructs

Three fragments of the promoter region of *EDS5H* were amplified using the High Fidelity Kit (Roche) with gene-specific primers designed to introduce *EcoR*I at the 5’ ends and *Nco*I sites at the 3’ ends of the fragments. The forward primers used for the amplification of EDS5H promoter fragments were: Pro500F, Pro1000F and Pro2000F resulting in fragments of 546 bp, 1057 and 1984 bp, respectively. The reverse primer for the EDS5H promoter fragments was ProE5H-R. The PCR fragments were cloned into the plasmid pCAMBIA 1303 (www.CAMBIA.org) from which the fragments of the promoter CaMV 35S and LacZ alpha had been removed.

The ORF of EDS5H was amplified by High-fidelity RT-PCR using the Omniscript RT-PCR system (Qiagen) and the forward primer Nco-E5H introducing a *Nco*I site as well the reverse primer E5H-Mycs introducing a triple cMyc-tag and the restriction sites *Sma*I and *Nde*I. The PCR fragment was cloned into pGEM-T Easy Vector (Promega) in the *Nco*I and *Nde*I sites. The clone was sequenced and the *Nco*I-*Sma*I fragment that included EDS5H::3xcMyc-tagg was then cloned into pCAMBIA 1304 (www.CAMBIA.org) from which the GUS and GFP genes had been removed.

### Construction of plasmid for overexpressing of *EDS5H*

The cDNA of *EDS5H* was amplified by RT-PCR using the forward primer E5H-For and the reverse primer E5H-Rev and was sub-cloned into pGEM-T Easy Vector (Promega). The cDNA was digested with *Eco*RI, blunted, and cloned into pART7 [33]. Clones were selected based on the correct orientation of the cDNA between the CaMV35S promoter and OCS terminator. The cassette of CaMV35S::EDS5H::OCS was digested with *Not*I and cloned into the binary vector pART27 [33].

### Constructions of plasmids for protein domain swapping

The constructs for the peptide-swapping experiments were designed to include the triple cMyc-epitope tag (3xcMyc-tag). Therefore, a fragment of 3xcMyc-tag was amplified using the synthesized oligonucleotide E5H-Mycs as template. The PCR fragment included a *Not*I site at 5’-end and a *Sma*I and *Nde*I sites at 3’-end and 4 alanine residues upstream of cMyc-tag (forward primer: For-Ala-Myc; reverse primer: Rev-Myc-PCS). The 3xcMyc-tag was cloned into pGEM-T Easy vector at corresponding sites.

*EDS5* and *EDS5H* genes were swapped at the *Nde*I site of *EDS5* (281 bp downstream of ATG). The *Nde*I site was introduced into *EDS5H* by changing of a nucleotide T to A (336 bp downstream of ATG) that caused no change in amino acid residues. Therefore, the N-terminus of each protein was designed to include *Nco*I and *Nde*I as cloning sites. The fragments were amplified by RT-PCR using forward primer Nco-EDS5, reverse primer EDS5-Nde for *EDS5*, and forward primer Nco-E5H, reverse primer E5H-Nde for *EDS5H*. The C-terminus of each protein was designed to include *Nde*I and *Not*I as cloning sites. The fragments were amplified using forward primer Nde-eds5, reverse primer EDS5-Rev for *EDS5*, and forward primer Nde-e5h, reverse primer E5H-Swap-Rev for *EDS5H*. The swapped domains of either EDS5::EDS5H or EDS5H::EDS5 were constructed in pBluescript (Stratagene).

The constructs were placed upstream of 3xcMyc-tag with *Nco*I and *Not*I sites. The cassettes of the swapped peptide domains and 3xcMyc-tag were then cloned into pCAMBIA 1304 (www.CAMBIA.org). The *GFP* and *GUS* reporter genes of the binary vector were replaced with either *EDS5::EDS5H*::3xcMyc-tag or *EDS5H::EDS5*::3xcMyc-tag cassettes at *Nco*I and *Pml*I sites.

### Immunolabelling and fluorescence microscopy

For immunolabelling, plant tissue was prepared according to the protocol described in [34]. The PEG-embedded tissue was sectioned into 4–5 μm-thick slices and placed on poly-L-lysine-covered slides. The slides were incubated in PBS for 10 min in order to remove the PEG from the section. Each slide was blocked for free aldehyde by incubation with 0.1 M NH_4_Cl (in PBS) for 5 min, and washed for 5 min with PBS. The unspecific binding sites were blocked by incubation with 5 % BSA (in PBS) for 30 min. Slides were then incubated overnight with primary antibody solution (mouse anti-cMyc diluted in 5 % BSA/PBS in ratio 1:500) at 4 °C, washed three times with 0.1 % BSA (in PBS) for 10 min each, and once with 1 % BSA (in PBS) for 10 min. The secondary antibody used was goat anti-mouse-IgG conjugated with Alexa Fluor 488 or goat anti-rabbit-IgG conjugated with Alexa Fluor 568, diluted 1:500 in 5 % BSA (in PBS). Slides were placed in a humid chamber, incubated with secondary antibody for 2 h at 37 °C. After washing with PBS (4x10 min, samples were kept at 4 °C in the dark. Fluorescent signals were recorded with a BioRad MCR 1024 Kripton-Argon confocal microscope using an excitation wavelength of 488 nm with an emission filter 522 DF32, or an excitation wavelength of 568 nm with emission filters 605 DF32 and 585 EFLP.

### GUS-assays and determination of SA

GUS assays and the determination of free and conjugated SA were performed as described previously [4, 35].

## Additional file

Additional file 1:
**List of primers.**
