# Supplementary material for: Localization and expression of EDS5H a homologue of the SA transporter EDS5
Source: BMC Plant Biol. 2015 Jun 9;15:135. doi: 10.1186/s12870-015-0518-1 (PMC4459457; doi:10.1186/s12870-015-0518-1)
Supplement: Additional file 1: — List of primers. [file 12870_2015_518_MOESM1_ESM.pdf]

Supplemental data

Supplemental Fig. 1. List of primers

Primer name	Sequence (5'-3')
75UpStream	ATC AAC GCA ACG CCG AGA AGC-
150UpStream	CAA TCA TTA GAC CGA ATG CTT AC
50DownStream	GAT CTA CAA CTG AAT GAA GCT G
100DownStream	CTT TTC TAT TCA GTC GAA TAC
200DownStream	CAG GGG AAT CAC AGG AGT TG
250DownStream	GCG GAC CGT GAT ACT TTA TC
300DownStream	GAG AGC TAG AGG ACA TGA TTC
JL-202	CAT TTT ATA ATA ACG CTG CGG ACA TCT AC
GSP1	CAT CAT CGT GAC TCC ACA AG
GSP2	CCT TCA TTC CCG AGT TTC AG
GSP3	CTG TGA GTA CTT GGA GTG ATG
GSP4	GCC CCT GTC TTT ATA ACT ATG
E5H-sens-For	CTC GAG ATC AAC GCA ACG CCG AGA AG
E5H-sens-Rev	GGT ACC TCT ACT TCT GCT TCC ACT TC
E5H-anti-For	GGA TCC ATC AAC GCA ACG CCG AGA AG
E5H-anti-Rev	ATC GAT TCT ACT TCT GCT TCC ACT TC
Pro500F	GAATTCACTGCGGACTAAATGATCAAG
Pro1000F	GAATTCGTCATTCCACTGATGGGGATTC
Pro2000F	GAATTCTGGCGAAGATGTGGTACTATC
ProE5H-R	CCATGGCACCAAACCCCTGAAAAA
Nco-E5H	CAG GCC ATG GGA CAA ATT CAA TGC AAA ACC CTA
E5H-Mycs	CCT TAA GCA TAT GCC CGG GCT AAA GGT CCT CCT CGG AGA TAA GCT TCT GCT CAA GGT CCT CCT CGG AGA TAA GCT TCT GCT CAA GGT CCT CCT CGG AGA TAA GCT TCT GCT CCG CAG CTT TCA CTT TCT CAG
E5H-For	ATC AAC GCA ACG CCG AGA AG
E5H-Rev	TTT CTT GTG AGG TAT ACG GTG
E5H-Mycs	CCT TAA GCA TAT GCC CGG GCT AAA GGT CCT CCT CGG AGA TAA GCT TCT GCT CAA GGT CCT CCT CGG AGA TAA GCT TCT GCT CAA GGT CCT CCT CGG AGA TAA GCT TCT GCT CCG CAG CTT TCA CTT TCT CAG
For-Ala-Myc	AGC GGC CGC AGC TGC GGA GCA GAA GCT T
Rev-Myc-PCS	AGC ATA TGC CCG GGC TAA AG
Nco-EDS5	CAG GCC ATG GGA CTA ATC AAA TCC CAA AGA TTG
EDS5-Nde	CCT TAA GCC ATA TGC TCT GTT TCA CCA GAT C
Nco-E5H	CAG GCC ATG GGA CAA ATT CAA TGC AAA ACC CTA
E5H-Nde	CCT TAA GCA TAT GCT CTG AGT CGC CAA ATC
Nde-eds5	GTG AAA CAG AGC ATA TGG GAA CAG
EDS5-Rev	TTG CGG CCG CAA TGG ATT TAA TCT TCT C
Nde-e5h	TCA GAG CAT ATG GGG ACA GAT GAA AGA GAT C
E5H-Swap-Rev	TTG CGG CCG CAG CTT TCA CTT TCT C
